# Non-Linear Periodization for General Fitness & Athletes

**DOI:** 10.2478/v10078-011-0057-2

**Published:** 2011-10-04

**Authors:** Steven J. Fleck

**Affiliations:** 1 Sport Science Department, Colorado College, Colorado Springs, Colorado, U.S.A.

**Keywords:** periodization, linear, nonlinear, youth

## Abstract

Periodization of resistance training or planned changes in training volume and intensity are used to maximize strength and fitness gains. Several types of periodized resistance training plans have been developed. The most common of these plans is linear also termed classic or strength/power periodization and nonlinear periodization. The biggest difference between these two types of training plans is with nonlinear periodization changes in training volume and intensity are made more frequently. The most common type of nonlinear periodization is daily nonlinear periodization where substantial changes in training intensity and volume are made from one training session to the next training session. Periodized resistance training does result in greater strength gains than non-periodized programs. While both linear and nonlinear periodization plans result in significant strength and fitness gains some research indicates greater strength gains with daily nonlinear periodization.

## Introduction

Training periodization has long been used by athletes and coaches in an attempt to maximize fitness gains and physical performance. More recently fitness enthusiasts and personal trainers have also begun to utilize periodized training plans. Although all types of training (aerobic, sport specific, interval training) can be periodized here only periodization of resistance or weight training will be considered. Periodization of resistance training refers to planned changes in the acute training program variables of exercise order, exercise choice, number of sets, number of repetitions per set, rest periods between sets and exercises, training intensity, training volume, and number of training sessions per day in an attempt to bring about continued and optimal fitness gains. The main goals of periodized training are optimizing training adaptations during both short periods of time (e.g. weeks, months) and long periods of time (e.g. years, an entire athletic career). Some periodized plans also have as a goal to peak physical performance at a particular point in time, such as for a major competition. Another goal of periodized resistance training is to avoid training plateaus.

A large number of studies and a meta-analysis indicate periodized resistance training programs result in greater strength increases in both sexes, untrained individuals and trained individuals compared to non-varied programs or performing the same number of sets and repetitions per set for the entire training program (Rhea and Alderman 2004). Changes in the acute training program variables result in a virtually limitless number of possibilities and so a limitless number of both short- and long-term periodized training strategies. However, two major types of periodized resistance training: classic, linear or traditional strength/power periodization and nonlinear periodization have received the most attention from the sport science, coaching and fitness enthusiast communities.

### Types of Periodization

Long-term programs of classic, linear or traditional strength/power periodization programs begin with high volume-low high-intensity training and progress towards low-volume high-intensity training. Normally the entire training plan takes several months to complete. If training is continued the entire plan beginning with high-volume low-intensity training is repeated. Typically with linear periodization different training phases of different volumes and intensities last approximately 4–6 weeks. Thus 4–6 weeks of training may be spent predominantly training with a particular training zone, such as 8–10 or 1–3 repetitions per set. Different training phases in many linear plans have a specific training goal and name, such as hypertrophy, strength, strength/power and power. One goal of many linear periodization programs is to peak or maximize strength/power after the last training phase typically termed a power phase.

While with nonlinear periodization training intensity and volume are changed much more frequently (Fleck and Kraemer 2004; Kraemer and Fleck, 2007). In fact the most common type of nonlinear periodization is termed daily nonlinear periodization. In this type of periodization training zones are changed in successive training sessions that is a different number of repetitions per set are performed from training session to training session. The simplest pattern of daily nonlinear periodization uses three training zones, such as 4–6, 8–10 and 12–15 repetition maximum zones, with each training zone being used for one training session per week for a total of three training sessions per week. Nonlinear programs have also been developed where a particular training zone is utilized for one- or two-week periods before a change in training volume and intensity is made. Such programs are typically called weekly or biweekly nonlinear periodized plans.

No matter which type of periodized resistance training is used other changes than the number of repetitions per set can also be made. Number of sets per exercise can be increased or decreased to change total training volume. Different exercises can be performed as the training progresses. Typically this change involves removing single joint exercises while keeping multi-joint exercises as training progresses. In many training plans other changes in the type of resistance exercises are also made, such as variations of the Olympic lifts, are emphasized when a training phase emphasizes power. In many periodized plans changes in intensity and volume are utilized mainly for multi-joint or multi-muscle group exercises. Thus in many periodized plant single joint or single muscle group exercises are not periodized. Although all of these changes are used in periodized plans research has predominantly examined the effect of different numbers of repetitions per set, which is a change in intensity and volume, when comparing periodized training plans.

### Periodization Comparisons

Most research comparisons of periodization models are between daily nonlinear and linear periodization with training durations of between 9–15 weeks. Some comparisons show significantly greater strength gains with daily nonlinear peridoization in college-age males (Rhea et al. 2002; Monteiro et al. 2009). While other comparisons show nonsignificant differences between the two types of peridoization, but favor nonlinear (Kok et al. 2009; Prestes et al. 2009) or the linear periodization (Bufford et al. 2007; Hartman et al. 2009; Hoffman et al. 2009) for percentage gains in maximal strength. Most comparisons involved healthy young males and females with limited or no resistance training experience, while one study trained collegiate American football players (Hoffman et al. 2009). So research training experienced weight trainers and athletes is needed. The above studies indicate daily nonlinear periodization is at least as effective or possibly more effective than the linear periodization for maximal strength gains.

A limited number of studies indicate motor performance and power increases are not significantly different between daily nonlinear and linear periodization (Hartman et al. 2009; Hoffman et al. 2009). Additionally, body mass and body composition changes with these two types of peridoization are similar and do not significantly change during the training durations investigated (Rhea et al. 2002; Bufford et al. 2007; Hoffman et al. 2009; Kok et al. 2009; Monteiro et al. 2009; Prestes et al. 2009). However, all but one of these studies (Rhea et al. 2002 used plesmography) used skinfolds to estimate body composition which may not be sensitive enough to determine small changes in body composition or differences between training programs.

Comparisons of weekly and biweekly nonlinear to linear periodization also show little difference in fitness gains between periodization plans. For example, a comparison of biweekly nonlinear periodization, linear periodization and a non-varied training program (3 sets of 6 repetitions) has been performed. The results showed all training types significantly increased maximal strength, vertical jump and fat-free mass, with no significant differences between training types (Baker, Wilson and Carlyn 1994). Collectively the above comparisons indicate nonlinear and linear periodization show little difference in body composition or motor performance changes.

### Flexible Nonlinear Periodization

Flexible nonlinear periodization is a relatively new type of periodization. Flexible nonlinear periodization uses the nonlinear training model but allows changes in training based upon the readiness of a trainee to perform a specific training zone. The decision to change the planned training zone for a specific training session is made using several pieces of information. A test, such as a maximal vertical jump, standing long jump or medicine ball throw, can be performed immediately prior to a training session to help determine the readiness of a trainee to perform a specific training zone. The beginning sets of the first few exercises in a training session can also be monitored to help determine the physical readiness of a trainee to perform a specific training session.

For example, if a standing long jump is performed immediately prior to a training session and the trainee cannot achieve at least 90% of their previous maximal standing long jump, the trainee may be fatigued. Similarly fatigue is indicated if an individual could previously perform 10 repetitions of an exercise with a specific resistance and at the start of a training session can only perform seven repetitions with this resistance. The fatigue or other physiological factor, such as delayed onset muscle soreness, could be due to previous resistance training sessions or other types of training (interval training, sports skill training) being performed as part of the total training program. Psychological stress due to work or any other factor could also prevent performing up to previously demonstrated abilities. No matter what the reason in this example if a moderate-intensity moderate-volume (4 sets of 10–12 repetitions) training zone was scheduled to be performed the training zone would be changed to a different zone (3 sets of 12–15 repetitions).

It is also possible to change from a low-intensity high-volume training zone to a higher intensity and lower volume zone. For example, a standing long jump is performed and 100% of the best standing long jump is achieved or sets of 8–10 repetitions are planned, but the trainee achieves 12 repetitions per set in the first exercise of a training session. In this case rather than continuing with a training zone of 8–10 repetitions a higher intensity zone (4–6 repetitions) may be performed because fatigue is not indicated and it appears the trainee is ready to train at a high intensity. Flexible daily nonlinear periodization and training zone changes have been previously extensively discussed (Kraemer and Fleck 2007).

To date, little research has been performed concerning flexible nonlinear periodization. A variation of this type of periodization has been employed to maintain and increase physiological markers in collegiate Division I soccer players throughout a 16-week season (Silvestre et al. 2006). Resistance training sessions were changed to meet the players readiness to perform a specific type of training session based upon the strength and conditioning coaches subjective evaluation and heart rates during soccer practice sessions and games. The flexible nonlinear periodized program resulted in the maintenance of vertical jump ability, short sprint ability and maximal oxygen consumption throughout the season. However, significant increases in total lean tissue, leg lean tissue, trunk lean tissue, total body power (17% increase in repeat push press power) and lower body power (11% increase in repeat squat jumps followed by a short sprint) were shown pre - to post-season. This study did not compare flexible nonlinear periodization to a different type of training. However, the results indicate the flexible nonlinear periodization did maintain or increase fitness markers throughout a soccer season.

A comparison of a flexible daily nonlinear to nonlinear periodization indicates flexible nonlinear periodization offers some advantages (McNamara and Stearne 2010). Students in a college weight training class performed either a flexible nonlinear or planned (had to perform the planned training session on a specific day) nonlinear periodized program two times per week for 12 weeks. The individuals performing the flexible nonlinear program could choose prior to a training session which of three training zones (10, 15, 20 repetitions per set) they would perform. However, at the end of the 12 weeks of training trainees in the flexible nonlinear program had to perform the same number of training sessions in each training zone as the planned nonlinear program.

Pre- to post-training one repetition maximal (1 RM) chest press ability and maximal standing long jump ability significantly increased with both training plans with no significant difference shown between plans. However, 1 RM leg press ability increased significantly more with the flexible nonlinear program. These results indicate the flexible nonlinear periodization plan did not result in a significant greater increase in upper body strength, but did significantly increase lower body strength to a significantly greater degree. Although little research has been performed on flexible nonlinear periodization this type of training plan appears promising.

Periodized resistance training does result in greater fitness increases than non-periodized programs. Nonlinear periodization results in similar fitness gains or possibly even greater fitness gains than linear periodization. While flexible nonlinear periodization has received little study by the sports science community it appears to be a promising periodized type of training. Thus coaches and fitness enthusiasts can use nonlinear and flexible nonlinear periodization plans with confidence that these types of periodization will result in significant fitness gains.
